# Validating electrical stimulation through the somatosensory system: a perspective on mismatch negativity and auditory prostheses

**DOI:** 10.3389/fnhum.2026.1912789

**Published:** 2026-10-06

**Authors:** Sergei Tugin, Polina Pavlova, Natalia Karpova, Boriss Sagalajev

**Affiliations:** 1 Research Center for Genetics and Life Sciences, Sirius University of Science and Technology, Sirius Federal Territory, Russia; 2 Centre for Research on Talent Development, Sirius University of Science and Technology, Sirius Federal Territory, Russia

**Keywords:** cochlear implant, electrical stimulation, event-related potentials (ERP), mismatch negativity (MMN), predictive coding, somatosensory system

## Abstract

Natural touch engages specialized mechanoreceptors, while electrotactile stimulation bypasses them, directly depolarizing a mixed and non-uniform population of afferent axons. Whether this difference in peripheral encoding affects the brain predictive processing remains an open empirical question. The mismatch negativity (MMN), an established electroencephalography (EEG) marker of automatic change detection, provides a powerful tool to address this question without perceptual confounds. Somatosensory studies comparing MMN responses to natural versus electrical stimulation within the same healthy individuals can identify candidate mechanisms for testing in the auditory domain, where such designs are inherently not feasible. In particular, they can help disentangle device effects from deafness-related confounds in cochlear implant (CI) users. Existing somatosensory evidence in healthy subjects suggests that electrotactile stimulation elicits mismatch responses with less bilateral integration than natural touch, though this evidence remains limited to a single magnetoencephalography (MEG) study using an oddball paradigm. CI studies are more extensive and show feature-specific MMN deficits. However, they also rely on oddball or multi-feature paradigms that conflate prediction error with sensory adaptation. This Perspective therefore encourages the adoption of alternative MMN paradigms (roving, omission, or local–global) to determine whether electrical stimulation truly engages the brain’s predictive machinery. With emerging multimodal stimulators, the somatosensory system offers a valuable *p*latform to test whether electrical stimulation reproduces natural predictive processing—insights that can improve both tactile neuroprostheses and CI devices.

## 1 Why natural and electrical stimulation cannot be assumed equivalent

The processing of environmental information reliably evokes characteristic responses in the higher nervous system. However, far less is known about the corresponding responses when information is delivered not via sensory receptor activation, but rather via direct electrical stimulation of primary afferents. Auditory research has long benefited from systems that enable natural sound delivery with precise control over pitch, volume, and timing. Somatosensory research, by contrast, lacks widely available mechanical stimulators capable of delivering reproducible natural touch with precise temporal locking. As a result, many somatosensory studies use electrotactile stimulation—delivered via skin surface electrodes—as a practical alternative (Alouit et al., 2025).

At first glance, this substitution appears unproblematic. Electrical stimulators are inexpensive, easy to synchronize with electroencephalography (EEG) recording, and offer millisecond-accurate control over pulse timing, duration, and intensity. However, this apparent convenience masks several critical issues. The first is a source of EEG noise: the electrical pulse itself generates a large stimulus artifact that couples directly into EEG electrodes, often saturating amplifiers and obscuring early cortical responses (Caldwell et al., 2020). The second is a mechanistic difference rather than measurement noise: natural and electrical stimuli engage different mechanisms of spike initiation. Natural touch activates specialized mechanoreceptors in the skin—Meissner, Pacinian, Merkel, and Ruffini endings—each with distinct adaptation properties, response thresholds, and central projections (Abraira and Ginty, 2013). Electrotactile stimulation bypasses mechanoreceptor transduction, directly depolarizing afferent axons via voltage-gated sodium channels (Yang et al., 2018). The population of afferents recruited by surface electrical stimulation is neither uniform nor exclusively cutaneous: depending on electrode geometry, current amplitude, and depth of current penetration, it may engage a mixture of cutaneous mechanoreceptor afferents, nociceptive fibers, and deeper proprioceptive afferents, with lower intensities preferentially recruiting larger-diameter axons closest to the electrode. The third is likewise a mechanistic difference: conventional electrical stimulation induces overly synchronous firing of primary afferents, resulting in an unpleasant tactile sensation known as paresthesia (Sagalajev et al., 2024). This difference in the stimulation approach means that electrically evoked event related potentials (ERP) may not faithfully represent natural somatosensory processing (Alouit et al., 2025). Findings based on electrotactile stimulation, therefore, require cautious interpretation. Without side-by-side comparisons of natural versus electrically evoked somatosensory ERP, we risk conflating two distinct phenomena, in effect confusing apples (natural touch) with oranges (electrical stimulation).

Yet electrotactile stimulation offers an important advantage that should not be dismissed: it can be applied non-invasively in individuals without sensory deficits. In hearing research, by contrast, electrical stimulation is largely restricted to patients with a cochlear implant (CI), whose hearing loss introduces disease-related confounds that are difficult to separate from the stimulation effects (Rance and Starr, 2015). The somatosensory field is therefore well positioned to bridge this gap, offering an opportunity to validate how electrically evoked ERPs differ from natural ERPs in healthy participants. The central argument of this perspective rests on three pillars: (a) natural touch and electrical stimulation differ at the peripheral level, where mechanoreceptor activation and direct axonal depolarization engage distinct encoding mechanisms; (b) it remains an open empirical question whether these peripheral differences propagate to alter cortical processing, or whether higher-level prediction machinery operates similarly regardless of input origin; and (c) resolving this question in the somatosensory system has direct translational significance for CI research, where device-related and deafness-related effects cannot *otherwise be separated.* Concretely, if somatosensory comparisons reveal that direct axonal stimulation systematically disrupts specific cortical computations (e.g., bilateral integration or temporal prediction), this would generate a strong hypothesis that analogous CI deficits may reflect device-related limitations rather than deafness alone. Such findings would motivate direct testing of this hypothesis in CI users and, if confirmed, would encourage targeted improvements in stimulation paradigms.

But what exactly should we measure to test whether peripheral differences in stimulation mode alter cortical processing? One powerful approach is to examine mismatch negativity (MMN), a well-established EEG marker of automatic change detection that arises when a rare “deviant” stimulus interrupts a sequence of repeated “standard” stimuli (Näätänen et al., 1978). MMN can test whether a sensory neuroprosthesis enables users to differentiate between inputs. Comparing electrical MMN (electrical deviant vs. electrical standard) to natural MMN (natural deviant vs. natural standard) reveals whether the brain can discriminate between events despite differences in input quality. Conversely, divergent responses would indicate that electrical stimulation disrupts discriminative capacity itself, not merely the subjective quality of sensation.

However, a stricter test than unimodal MMN is needed to determine whether electrical stimulation can reproduce natural-like predictive processing, not merely comparable discrimination. Specifically, this requires a cross-modal MMN assessment (electrical deviant vs. natural standard, or vice versa) using a within-subject design, where the absence of a cross-modal MMN would provide evidence that the two stimulation modes engage sufficiently similar predictive representations despite their different peripheral origins. Implementing such a task, however, is anything but trivial in EEG. Delivering cross-modal stimuli seamlessly for the subject requires a single stimulator capable of producing two fundamentally different kinds of input—natural and electrical—without introducing timing jitter, trigger uncertainty, or disruptive artifact. In the auditory field, such a device is impossible, because non-invasive natural sound and direct electrical stimulation cannot be combined in the same healthy individual. In the somatosensory field, however, such stimulators are not only possible but are now emerging (Figure 1) (Kirasirova et al., 2026).

**Figure 1:**
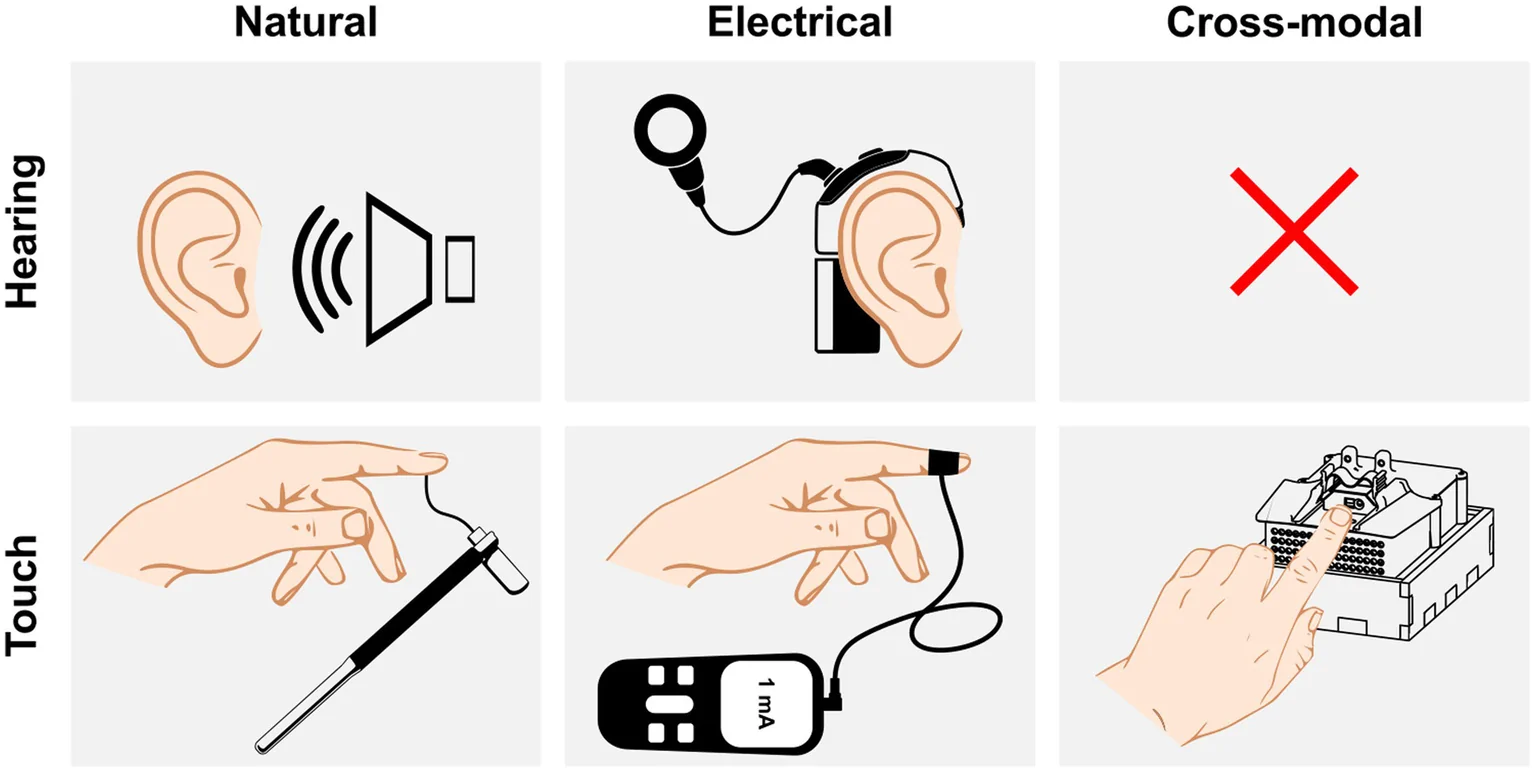
Cross-modal stimulation. Unlike auditory research, the somatosensory field allows direct comparison of natural and electrical stimulation within the same healthy individual using multimodal stimulators such as the one described by Kirasirova et al. (2026).

## 2 What MMN reveals about the brain’s prediction machinery

To apply cross-modal MMN to the natural-versus-electrical problem, we must first understand what MMN measures. That requires revisiting its discovery and the cascade of interpretations that followed.

The MMN is the well-studied automatic deviance-detection signal in human cognitive electrophysiology. It was first demonstrated by Näätänen et al. (1978), who reinterpreted what had been described as an “early selective attention effect” on the auditory ERP. They showed that a substantial part of that negative deflection in the ERP waveform (previously thought to reflect attentional processes) actually arose independently of attention to the stimuli (Näätänen et al., 1978). Specifically, subtracting the response to a frequent “standard” tone from the response to a rare “deviant” embedded in the same sequence yielded a negative-going difference wave, typically peaking between 100 and 250 ms after deviant onset, with a frontotemporal scalp maximum (Näätänen et al., 1978). Although this operational definition of MMN has remained remarkably stable, what has changed more substantively is what the MMN is believed to reflect. Over the four decades since its discovery, five major hypotheses have emerged. These hypotheses are presented in a specific sequence that mirrors both their historical development in the literature and their increasing degree of cognitive sophistication, moving from purely sensory mechanisms toward active inferential processes. Later hypotheses do not necessarily exclude earlier ones but rather supersede them by accounting for a broader range of empirical findings (Garrido et al., 2009).

### 2.1 Change detection hypothesis

The earliest account held that the MMN reflects the brain’s detection of a local physical change in the incoming sound, such as a shift in frequency or intensity. This mechanism does not require memory or prediction; it simply registers that the current stimulus differs from the immediately preceding one (Escera et al., 1998, 2003).

### 2.2 Adaptation hypothesis

A subsequent, more parsimonious account appealed to neural fatigue. Repeated exposure to the same sound reduces the response of sensory neurons tuned to that sound. When a different sound occurs, less adapted (or “fresh”) neurons respond more strongly. The MMN is, therefore, the difference in activity between these adapted and non-adapted neuronal populations. No active prediction or memory is needed, only the biological fact of neural fatigue (Jääskeläinen et al., 2004; May and Tiitinen, 2010).

### 2.3 Model adjustment hypothesis

As evidence accumulated that MMN could be elicited by violations of abstract regularities, a more cognitive account emerged. Unlike simple physical changes (e.g., a low tone replaced by a high tone), abstract regularities involve relationships between stimuli over time. For example, the brain can learn an alternating pattern (low, high, low, high). When an incoming sound violates such a learned pattern (such as hearing two high tones in a row) the brain registers a prediction violation. According to this view, the brain continuously builds and maintains an internal model of the environment. When an incoming stimulus does not fit this model, the model is updated. The MMN indexes this process of model adjustment, reflecting the brain’s active effort to revise its representation of environmental regularities (Schröger and Winkler, 1995; Winkler et al., 1996; Winkler and Czigler, 1998).

### 2.4 Novelty detection hypothesis

A refinement of the model-adjustment view proposed that the MMN signals the degree of surprise elicited by an event—that is, how strongly it violates a probabilistic prediction. Surprise differs from mere physical change: in a context where a change occurs predictably, the absence of that change may be more surprising than its presence. Surprise is an undirected quantity; it indicates that an event is unexpected but does not specify in which way the prediction was violated. Bayesian observer models have successfully predicted single-trial MMN responses based on stimulus surprise in hearing, vision, and touch (Tiitinen et al., 1994; Ostwald et al., 2012; Gijsen et al., 2021).

### 2.5 Prediction error hypothesis

The most current and computationally precise account comes from predictive coding theories. Under this view, the brain acts as a hierarchical inference machine that maintains internal generative models of its sensory environment and continuously updates those models against incoming evidence (Knill and Pouget, 2004; Friston and Kiebel, 2009). The MMN reflects the neural activity encoding prediction errors—that is, the difference between the actual input and the brain’s prior prediction. Unlike surprise, a prediction error carries directional information, indicating whether the actual event was, for example, higher or lower in pitch, or louder or softer than expected. This directional signal allows the brain to refine its predictions more precisely. The predictive coding framework implies a specific cortical processing stream, wherein higher areas generate predictions conveyed to lower areas via backward connections. These predictions are then compared with sensory input, and the resulting prediction error is used to adjust higher-level representations until the error is minimized (Friston, 2005). Computational models—composed of spiking excitatory and inhibitory neurons in a layered cortical architecture with distinct input, prediction, and error-signaling populations—reproduce many features of the MMN waveform, including its modulation by deviant probability and local transition statistics (Wacongne et al., 2011, 2012; Stefanics et al., 2018).

Across these five hypotheses a common thread emerges. Each describes, with increasing cognitive sophistication, how the brain registers that something in its sensory environment has changed (Fitzgerald and Todd, 2020; Gütlin et al., 2025; Batsunov et al., 2026). Notably, direct formal comparisons of the five major hypotheses using single-trial EEG data have shown that models based on the free-energy principle (unifying predictive coding, novelty detection, and model adjustment) provide a more plausible explanation of trial-by-trial MMN amplitude fluctuations than traditional adaptation or change detection models (Friston, 2010; Lieder et al., 2013). Therefore, the key takeaway is that the MMN provides a well-characterized, theory-rich measure of the brain’s active prediction machinery—the very machinery that must be engaged if electrical stimulation is to substitute for natural sensory input.

Although predictive coding currently provides the most influential framework, alternative interpretations based on adaptation remain actively discussed, and the relative contribution of each mechanism likely depends on paradigm and stimulus parameters. This dissociation between prediction error and adaptation is especially pertinent for the somatosensory MMN, whose status as a genuine prediction-error signal is more actively debated than its auditory counterpart. A recent comprehensive review (Grundei et al., 2025) examines whether the somatosensory MMN reflects true deviance detection or is largely attributable to stimulus-specific adaptation and refractoriness. This debate directly reinforces the case for the alternative paradigms discussed below: precisely because the adaptation confound may be more severe in somatosensory contexts, paradigms that control for it become a prerequisite for valid inference.

## 3 Oddball: the classical MMN paradigm

The oddball paradigm is the workhorse of MMN research due to its simplicity: a high-probability standard establishes a regularity, whereas a low-probability deviant violates it. However, this simplicity masks a conceptual ambiguity that becomes critical when comparing natural and electrical stimulation. Specifically, the deviant differs from the standard in two confounded ways. First, the deviant violates the established regularity. Even in the simplest case—where the same stimulus repeats over and over—the brain treats “repetition” itself as an abstract regularity (a rule that the next stimulus will be identical to the previous one). When a physically different deviant occurs, it breaks that rule, generating a prediction error. Second, the deviant benefits from release from sensory adaptation: standard-responsive neural populations are fatigued by repetition; deviant-responsive populations are fresher (May and Tiitinen, 2010). Thus, in the oddball paradigm, the MMN reflects a mixture of prediction error and release from adaptation, requiring control conditions to disentangle their contributions (Tiitinen et al., 1994; Näätänen et al., 2007).

### 3.1 Deviant-alone control

The deviant stimulus is presented in a separate block at the same low overall rate as in the oddball, but without intervening standards. If the deviant evokes a smaller response in this control than as a deviant in the oddball paradigm, the additional response in the oddball cannot be explained by stimulus-specific refractoriness alone, since the deviant stimulus is presented equally often in both conditions (Schröger and Wolff, 1996).

### 3.2 Many-standards control

The homogeneous standard stream is replaced with a stream of several physically distinct stimuli, each presented at the same low probability as the deviant of interest. Because no single stimulus is repeated often enough to drive substantial adaptation, a response specific to the deviant in the oddball paradigm that is absent in this control can be confidently attributed to regularity violation (prediction error) (Jacobsen et al., 2003).

## 4 Alternative MMN paradigms

The above control strategies for the oddball paradigm assume that standard and deviant belong to the same physical category. That assumption breaks down when comparing natural versus electrical stimulation, where the deviant–standard contrast inherits baseline sensory differences and creates a spurious “MMN” that could reflect unequal sensory encoding rather than genuine regularity violation. One solution, demonstrated in cross-sensory MMN studies, is to include a unimodal reference condition whose subtraction corrects for modality-specific differences (Ponton et al., 2009; Stekelenburg et al., 2018). While such subtraction would work in principle for natural versus electrical stimulation, the alternative paradigms described below circumvent subtraction entirely, yielding cleaner estimates of prediction error.

We review four such paradigms—roving, omission, local–global, and multi-feature—each addressing the natural-versus-electrical comparison in a different way. The omission paradigm provides the strongest test of equivalence by removing sensory input entirely. The roving paradigm offers clean baselines for each modality, enabling meaningful cross-modal comparisons. The local–global paradigm tests hierarchical prediction. And the multi-feature paradigm, while efficient, remains susceptible to adaptation and is therefore less suited for testing equivalence directly.

Before turning to each paradigm individually, two critical methodological principles must be stated. First, all of the paradigms described below share a common interpretive limitation when used in cross-modal designs: the absence of a mismatch response can only be interpreted as evidence of equivalence if the study employs an explicit equivalence or non-inferiority testing framework with adequate statistical *power.* Second, successful validation must be predefined before data collection, with clear inferential criteria established a priori. For basic proof-of-concept studies, validation may rest on MMN parameters alone (amplitude, latency, topography, source localization). However, for stronger inference—particularly for claims that have clinical implications or that concern shared predictive mechanisms—validation should require convergent evidence from at least two independent sources, such as behavioral discrimination measures and/or computational modeling. Without these safeguards, simply failing to reject a difference cannot be interpreted as evidence of equivalence.

### 4.1 Roving paradigm

The roving paradigm offers a critical advantage for the natural-versus-electrical comparison by eliminating stimulus-specific contributions to the MMN. In a conventional oddball, deviant and standard stimuli differ along some physical dimension (e.g., frequency, intensity, location), meaning any neural response difference could be driven by the physical properties of the stimuli themselves rather than by regularity violation. The roving paradigm circumvents this problem entirely. Here, a stimulus of a given type is presented several times in a row (e.g., 1 to 11 repetitions) before spontaneously “roving” to a new stimulus of a different type. The first presentation of a new stimulus is operationally defined as the deviant, while later presentations of the same stimulus serve as the standard. Crucially, deviant and standard are physically identical—they are the exact same stimulus presented at the same intensity, duration, and location. Thus, any difference in the evoked response cannot be attributed to differential activation of specific neuronal populations but must reflect the violation of an established regularity (Garrido et al., 2008).

Applied to the natural-versus-electrical problem, the roving paradigm could be implemented using either natural or electrical stimuli as the repeating “standard” that eventually roves. Because the physical stimulus is identical for deviant and standard in a given block, any observed MMN-like difference would directly index the brain’s ability to form a regularity representation from that stimulation type, unconfounded by peripheral response differences. These within-block comparisons thus provide a clean baseline for quantifying how well each modality supports predictive processing on its own. Crucially, they can then serve as reference points for interpreting between-block comparisons, making it possible to determine whether the brain treats the two input streams as interchangeable.

### 4.2 Omission paradigm

Whereas the roving paradigm controls for physical stimulus differences by making deviants and standards identical, the omission paradigm takes a different approach: rather than presenting a physically distinct deviant, an expected stimulus is occasionally omitted entirely from a regular train. Because there is no bottom-up sensory input at the moment of omission, any evoked response cannot reflect peripheral afferent activity. Instead, an omission-related response provides direct evidence for top-down expectation and predictive coding (Yabe et al., 1997; Andersen and Lundqvist, 2019).

For the natural-versus-electrical comparison, the omission paradigm offers a powerful control. In its unimodal application, comparing omission responses between natural and electrical stimulation (each in separate blocks) reveals whether each modality can independently establish temporal regularities sufficient to generate top-down predictions. Crucially, these unimodal baselines then enable cross-modal interpretation: omitting an expected electrical stimulus from a sequence of natural standards, or vice versa, makes it possible to determine whether the brain treats predictions derived from one input stream as transferable to the other.

### 4.3 Local–global paradigm

Whereas the roving and omission paradigms probe the brain’s ability to form temporal predictions based on stimulus repetition or expected occurrence, the local–global paradigm addresses a different level of predictive coding: hierarchical structure. Specifically, it dissociates low-level stimulus-specific adaptation from high-level, rule-based expectations by manipulating regularities at two distinct timescales simultaneously (Bekinschtein et al., 2009; Wacongne et al., 2011).

Each trial in the local–global paradigm consists of five sequential stimuli. The first four stimuli are always identical, either all A, or all B, while the fifth stimulus is either the same as the first four (AAAAA or BBBBB) or different (AAAAB or BBBBA). Across blocks of trials, the probabilities of these patterns are manipulated. In one block, the locally matching patterns (AAAAA and BBBBB) occur on 80% of trials, making them globally common, while the locally mismatching patterns (AAAAB and BBBBA) occur on 20% of trials, making them globally rare. In the other block, these probabilities are reversed: the locally mismatching patterns become common (80%) and the locally matching patterns become rare (20%). This reversal creates a dissociation. Locally, AAAAB (and BBBBA) are deviants because the fifth sound differs from the previous four. Globally, however, they may be either expected (when common) or unexpected (when rare). Correspondingly, the early local MMN to AAAAB (or BBBBA) persists even when these patterns are common, reflecting automatic, stimulus-driven change detection that is insensitive to global probability. In contrast, the late global effect is elicited by global deviants, regardless of whether they are locally standard (AAAAA or BBBBB when rare) or locally deviant (AAAAB or BBBBA when rare). Thus, the local–global paradigm dissociates sensory-based prediction errors (local) from rule-based prediction errors (global). When applied to the natural versus electrical comparison, the ideal outcome in a cross-modal design is the absence of both the local MMN and the global effect, which would be consistent with the brain treating electrical and natural stimuli similarly from basic change detection to abstract rule learning.

### 4.4 Multi-feature paradigm

Whereas the roving, omission, and local–global paradigms each address a specific confound in the oddball design (physical stimulus differences, bottom-up sensory drive, or hierarchical rule learning), the multi-feature paradigm targets a different problem: efficiency. The classic oddball paradigm is inherently inefficient for characterizing multidimensional sensory processing because each deviant type requires a separate recording block. The multi-feature paradigm solves this by interleaving multiple deviant types within a single alternating sequence. For example, to measure five deviants—frequency, duration, intensity, location, and omission—the sequence alternates each deviant with a standard: S, D₂, S, D₁, S, D₃, S, D₅, S, D₄… (where D₁–D₅ represent the five deviant types). Standards and deviants occur with equal overall probability (50% each), and each deviant type occurs equally often (10% with five deviant types). The sequence prevents consecutive repetitions of the same deviant category, avoiding stimulus-specific adaptation. Critically, MMN amplitudes obtained with this interleaved design are statistically equivalent to those recorded using traditional oddball blocks, yet the multi-feature paradigm records MMNs for multiple deviant types in a fraction of the time (Näätänen et al., 2004). This dramatic reduction in recording time can be critical in settings where participant fatigue, compliance, or limited session duration pose practical constraints.

The logic of the multi-feature paradigm has been extended further by incorporating multiple magnitudes of deviation for each deviant type—yielding, for example, in a study with 4 deviant types and 6 magnitudes each, 24 distinct deviant conditions within a single 90-min session (Pakarinen et al., 2007). Under these conditions, MMN amplitude increases monotonically with deviation magnitude and accurately predicts behavioral hit rates and reaction times, confirming that the paradigm captures fine-grained, ecologically valid discrimination profiles rather than merely reflecting physical stimulus properties.

For comparing natural versus electrical stimulation in the somatosensory domain, the multi-feature paradigm offers an advantage that neither the roving, omission, nor local–global paradigms address: simultaneous assessment of feature-specific effects within a single feasible session. For example, if electrical stimulation selectively disrupts discrimination of certain features (e.g., duration vs. intensity) or impairs sensitivity to small versus large deviations, the multi-feature paradigm would reveal this as a feature-by-modality interaction. However, recent evidence suggests that the multi-feature paradigm may substantially underestimate the contribution of adaptation to the MMN, and genuine prediction-error-related responses for some features (e.g., intensity) may be absent altogether (Widmann et al., 2026). Thus, while efficient for profiling discriminative capacity across features, the multi-feature paradigm may not provide a pure measure of prediction error. In contrast, the roving, omission, and local–global paradigms address what kind of prediction error survives the natural-versus-electrical contrast (temporal, purely top-down, or hierarchical) with better isolation from adaptation confounds. Together, these alternative paradigms offer a complementary toolkit for dissecting the nature and extent of predictive coding deficits when natural sensory input is replaced by electrical stimulation.

## 5 MMN in hearing

The CI represents one of the most successful neuroprostheses, yet it remains fundamentally limited by two principal constraints. First, poor spectral selectivity arises because electrical current spreads widely within the cochlea’s perilymph and endolymph, recruiting tonotopically disparate spiral ganglion neurons beyond the intended frequency channel. Second, direct electrical depolarization of primary afferent neurons leads to a compressed dynamic range: typically only 10–30 dB between threshold and uncomfortable loudness, while natural hearing affords approximately 120 dB. This compression occurs because electrical stimulation bypasses the natural compression mechanisms of the cochlea, including the electromotility of outer hair cells and the heterogeneous ribbon synapses of inner hair cells (Moser et al., 2023). These limitations manifest behaviorally as difficulty understanding speech in the presence of competing sound sources, and as profoundly degraded music perception, particularly for pitch-based features (Wolf et al., 2022).

Against this backdrop, the MMN has emerged as an objective, attention-independent tool for probing how well CI users’ central auditory systems can automatically detect changes in sound features. However, as argued in previous sections, interpreting MMN findings in CI users requires careful attention to paradigm choice, given that the two most widely used paradigms for studying MMN in CI users—the classical oddball and the multi-feature paradigm—are both susceptible to adaptation.

### 5.1 Oddball studies

Across studies comparing CI users with normal-hearing (NH) controls using oddball paradigms, a remarkably consistent pattern emerges: the MMN can be elicited in CI users, but its properties are systematically degraded. Studies using speech or musical stimuli have repeatedly shown that CI users exhibit reduced MMN amplitudes, prolonged latencies, and shorter duration compared to NH controls (Timm et al., 2012; Zhang et al., 2013; Cai et al., 2020). Moreover, the MMN is detectable in only approximately half of CI users under certain conditions, whereas it is universally present in NH listeners (Zhang et al., 2013). However, one must interpret null MMN findings cautiously, as failure to detect MMN may simply reflect poor signal-to-noise ratio rather than a genuine deficit (Heidekrüger et al., 2019). With this caveat in mind, MMN absence within the CI population is not random but instead reflects clinically meaningful individual differences. Multiple studies have shown that MMN is detectable only in CI users with good speech perception abilities, whereas poor performers often show no identifiable MMN or have significantly reduced responses (Rahne et al., 2010; Zhang et al., 2025). This pattern extends to pitch discrimination as well: CI users with better behavioral pitch discrimination show larger MMN amplitudes and shorter latencies than poor performers (Cai et al., 2020). Notably, MMN responses to pitch changes are reliably observed at basal electrode pairs but are significantly reduced or absent at apical electrode pairs (Wagner et al., 2023). This apical deficit is particularly consequential given that CI electrode arrays typically cover only half of the cochlea, failing to fully reach the apical end and thereby undersampling the low-frequency region (Boyd, 2011). In contrast, intensity discrimination, which does not require fine-grained place coding, can be elicited consistently across the implanted electrode array (Wagner et al., 2023).

### 5.2 Multi-feature studies

Multi-feature paradigms have revealed that CI users retain some discriminatory capacity while showing feature-specific and age-dependent deficits. Automatic neural discrimination of intensity, timbre, and rhythm is largely preserved in both prelingually deafened adolescents and postlingually deafened adult CI users. Meanwhile, pitch discrimination is preserved only in the latter group (Timm et al., 2014; Petersen et al., 2015, 2020). The importance of implantation age is further supported by evidence that MMN amplitude to musical changes correlates inversely with the duration of profound deafness (Sandmann et al., 2010). While pre-implantation factors impose constraints, post-implantation plasticity may partly offset them: a longitudinal study of recently implanted users demonstrates significant improvement in neural discrimination of pitch and timbre within just 3 months of device switch-on. Notably, this neurophysiological improvement was not reflected in behavioral measures, highlighting a dissociation between automatic change detection and attentive discrimination (Seeberg et al., 2023). Reinforcing this dissociation, earlier work reported reduced MMN amplitudes for pitch in CI users compared to NH controls (Timm et al., 2014), whereas a later study found no significant MMN differences across any feature, despite persistent behavioral deficits (Petersen et al., 2020). Remarkably, MMN responses predict attentive sound discrimination ability in CI users with nearly 90% accuracy, supporting the MMN as a viable diagnostic tool for assessing music perception at the individual level (Haumann et al., 2023).

### 5.3 Additional components

Three paradigm types have been used to study additional ERP components in CI users: passive oddball (for N100, P200), active oddball (for P300), and music-syntactic oddball (for ERAN, N500).

The N100 and P200 are exogenous (stimulus-driven) components measured directly on the raw waveform. Both are reliably elicited in CI users but show characteristic abnormalities. N100 latency is delayed and its amplitude is reduced compared to NH controls, while P200 amplitude is also smaller, suggesting that even basic sensory encoding is compromised by electrical hearing (Timm et al., 2012).

In contrast, the later P300 component is endogenous (top-down) and attention-dependent, yet remarkably robust in CI users. It is detectable in nearly all individuals, with amplitudes often comparable to those of NH listeners. However, its latency increases with the difficulty of frequency discrimination, indicating that active attentional processing can compensate for degraded input at the cost of slower processing speed (Obuchi et al., 2012; Abrahamse et al., 2021).

Two additional components, the ERAN and the N500, resemble the MMN in that they are pre-attentive and are measured as difference waves. However, the crucial difference lies in what triggers each response. The MMN indexes physical deviance detection, meaning any change in low-level acoustic features such as pitch, intensity, or duration. In contrast, the ERAN and N500 index high-level processing of harmonic structure, responding to violations even when physical differences between chords are minimal or absent. For example, an unexpected chord that is physically similar to a regular chord will elicit little to no MMN, but will reliably trigger an ERAN if it breaks musical grammar (Koelsch, 2009). The ERAN peaks at 150–250 ms with a right-anterior distribution and reflects automatic detection of harmonic violations, whereas the later N500 indexes harmonic integration (Koelsch et al., 2000). Relative to NH listeners, CI users show significantly reduced amplitudes for both components. The ERAN also has a later onset and longer duration. Critically, only highly irregular chords (fifth position) reliably elicit either component, showing that CI users require more salient harmonic violations to generate a robust response (Koelsch et al., 2004), similar to the degradation observed for the MMN (Koelsch, 2009).

### 5.4 Implications

Together, oddball studies reveal partially preserved auditory function in CI users: basic sensory encoding (N100, P200) is impaired, with delayed latencies and reduced amplitudes; automatic deviance detection (MMN) is often reduced or absent, particularly for pitch-based changes; active attention-driven discrimination (P300) continues largely intact, though with slowed processing speed; and higher-order syntactic processing (ERAN, N500) remains detectable but requires more salient harmonic violations than in NH listeners. Notably, multi-feature studies indicate that MMN deficits in CI users are often feature-specific rather than universal. While intensity and certain timbre discriminations can be relatively preserved, sometimes showing no significant group differences from NH peers, pitch discrimination remains more vulnerable. This discrepancy suggests that oddball designs may overestimate hearing deficits and underestimate CI benefits (Table 1). Furthermore, while the multi-feature paradigm appears more sensitive to preserved feature discriminations, it shares the oddball’s susceptibility to adaptation, necessitating alternative paradigms (roving, omission, local–global) to isolate genuine prediction error in future studies.

**Table 1:** Comparison of auditory event-related potential (ERP) components between cochlear implant users and normal-hearing controls.

Type	Component	Time (ms)	Paradigm	Stimulus	A	L	EEG studies
ERP	N100	100–200	Oddball	Cornet	↓	(↑)	Timm et al. (2012)
				Consonants	↓	↑	Zhang et al. (2025)
ERP	P200	180–240	Oddball	Cornet	(↓)		Timm et al. (2012)
				Consonants	↓	↑	Zhang et al. (2025)
ERP	P300	~300	Oddball	Chords | timbre	↓		Koelsch et al. (2004)
				Pure tones	(↓)	(↑)	Obuchi et al. (2012)
				Pure tones	ns	ns	Abrahamse et al. (2021)
				Consonants	ns	ns	Abrahamse et al. (2021)
Δ ERP	MMN	100–250	Oddball	Vowels	(↓↑)		Salo et al. (2002)
				Chords | timbre	↓		Koelsch et al. (2004)
				Consonants | vowels	(↓)		Rahne et al. (2010)
				Cornet	↓	↑	Timm et al. (2012)
				Pure tones		↑	Obuchi et al. (2012)
				Timbre	↓	ns	Zhang et al. (2013)
				Pure tones | noise	X	X	Heidekrüger et al. (2019)
				Complex tones	(↓)	(↑)	Cai et al. (2020)
				Electrode Pairs	(↓)	(↑)	Wagner et al. (2023)
				Consonants	↓	↑	Zhang et al. (2025)
			Multi-feature	Music	(↓)	(↓↑)	Sandmann et al. (2010)
					(↓)	(↑)	Timm et al. (2014)
					(↓)	(↓↑)	Petersen et al. (2015)
					ns	ns	Petersen et al. (2020)
					(↓)	(↑)	Seeberg et al. (2023)
					(↓)		Haumann et al. (2023)
Δ ERP	ERAN	150–400	Oddball	Chords | timbre	(↓)		Koelsch et al. (2004)
Δ ERP	N500	500	Oddball	Chords | timbre	(↓)		Koelsch et al. (2004)

Findings are based on oddball and multi-feature paradigms. A, amplitude; L, latency; ↑, increase; ↓, decrease; ↓↑, bidirectional changes; (), depends on stimulus parameters and patient background; ns, non-significant; X, strong electrical artifacts.

The heterogeneity in MMN findings can be attributed to several factors. The first is stimulus feature, where pitch-based deviance consistently yields the most degraded MMN, whereas intensity, timbre, and rhythm discriminations are often relatively preserved (Timm et al., 2012; Zhang et al., 2013; Petersen et al., 2020; Wagner et al., 2023). Paradigm selection introduces systematic bias, as the adaptation-susceptible oddball tends to report larger deficits than the multi-feature paradigm (Näätänen et al., 2004; Widmann et al., 2026). Deviance magnitude further modulates detectability, with larger standard–deviant differences more likely to elicit a measurable MMN—particularly in CI users, who require more salient violations (Schröger and Winkler, 1995; Koelsch et al., 2004). Participant and implant characteristics—age at implantation, duration of deafness, prelingual versus postlingual onset, residual hearing, electrode insertion depth, and stimulation strategy—contribute substantial uncontrolled variance (Petersen et al., 2015; Seeberg et al., 2023; Boyd, 2011; Heidekrüger et al., 2019). Signal-to-noise ratio and artifact handling are especially critical, as electrical stimulation artifacts can contaminate the EEG and low trial counts reduce power; failure to detect an MMN may reflect an inadequate ratio rather than a genuine deficit (Caldwell et al., 2020). Finally, MMN analysis choices—peak versus mean amplitude measurement, electrode and latency window selection, and criteria for declaring MMN presence—vary across studies and can yield divergent conclusions from comparable data (Fitzgerald and Todd, 2020; Widmann et al., 2026). Recognizing these dimensions suggests that apparent inconsistencies in the CI MMN literature are largely methodological rather than biological.

## 6 MMN in touch

A striking asymmetry exists between the auditory and somatosensory fields regarding the study of the MMN elicited by electrical stimulation. In hearing research, electrical stimulation is delivered exclusively to CI users, generating a substantial literature with established clinical utility. In stark contrast, the literature on the MMN elicited by electrotactile stimulation is remarkably sparse.

Several studies have successfully elicited somatosensory MMN using electrotactile stimulation in healthy participants and have characterized its modulation by stimulus probability, interstimulus interval, and aging (Shinozaki et al., 1998; Akatsuka et al., 2005; Restuccia et al., 2009; Strömmer et al., 2014; Zhao et al., 2014). However, direct within-subject comparison with natural mechanical touch has only recently emerged (Hautasaari et al., 2019; Kirasirova et al., 2026). This scarcity is not due to lack of clinical relevance, as somatosensory neuroprostheses and spinal cord stimulators would benefit from objective measures of discriminative capacity. Rather, the paucity reflects a fundamental methodological bottleneck: the absence of widely available tactile stimulators capable of delivering both natural and electrical stimulation with precise temporal locking and minimal artifact.

We recently described a custom-built stimulator that overcomes this barrier (Kirasirova et al., 2026). Such devices remain commercially rare, forcing other labs to rely on in-house solutions of their own (Hautasaari et al., 2019). What limited evidence does exist comes from a single oddball study utilizing magnetoencephalography (MEG). Hautasaari et al. (2019) compared somatosensory mismatch responses (MMR) elicited by natural versus electrotactile stimulation, finding that both produced early activation of the contralateral primary (S1) and later activation of the contralateral secondary somatosensory cortex (S2). Critically, only natural stimulation elicited bilateral S1 activation during the late MMR window, suggesting that it engages more extensive integrative processing across hemispheres than electrotactile stimulation (Table 2). This finding suggests that while electrotactile stimulation can generate a MMR, the pattern of bilateral engagement may differ from that observed with natural touch, a conclusion that awaits confirmation from additional direct comparisons.

**Table 2:** Comparison of mismatch responses between natural and electrotactile stimulation in healthy participants.

MEG study	Stimulation	Cortex	Side	Time window	
				40–58 ms	110–185 ms
Hautasaari et al. (2019)	Natural	S1	Ipsi	ns	Deviant > Standard
			Contra	Deviant > Standard	Deviant > Standard
		S2	Ipsi	ns	ns
			Contra	ns	Deviant > Standard
	Electrical	S1	Ipsi	ns	ns
			Contra	Deviant > Standard	ns
		S2	Ipsi	ns	ns
			Contra	ns	Deviant > Standard

The table is based on a single magnetoencephalography study by Hautasaari et al. (2019). S1, primary somatosensory cortex; S2, secondary somatosensory cortex; ns, non-significant.

Translated to the auditory field, this suggests that similar hemispheric asymmetries in CI users may arise from CI limitations (e.g., due to poor spectral selectivity) rather than from hearing deficits. Consistent with this interpretation, MMN studies in CI users have reported abnormal hemispheric lateralization of MMN responses to musical features (Timm et al., 2014; Petersen et al., 2015). However, the translational step from somatosensory to auditory findings must be made with care. Receptor physiology, peripheral coding, cortical organization, temporal processing, and bilateral integration all differ between touch and hearing, and these differences may limit the generalizability of somatosensory results to CI research. Some principles are likely to transfer more readily than others. General consequences of direct axonal stimulation—such as compressed dynamic range and overly synchronous firing—have been documented in both auditory and somatosensory electrical stimulation. However, their extent and functional consequences depend critically on the neural interface, target tissue, and stimulation parameters (Sagalajev et al., 2024; Hughes and Laurello, 2017; Zeng et al., 2002; Runge-Samuelson et al., 2004; Menia and Van Doren, 1994). Thus, while these effects may generalize more readily than modality-specific cortical principles, they should not be assumed to transfer uniformly or with identical functional impact across modalities. Somatosensory findings should therefore be viewed not as direct evidence for CI mechanisms, but as a hypothesis-generating bridge: they can identify candidate mechanisms to be tested empirically in the auditory domain before being accepted as explanations for CI outcomes.

## 7 Conclusion

Natural and electrical stimulation may not evoke identical cortical responses, yet electrotactile stimulation is often substituted for natural touch, necessitating cautious interpretation. The MMN component constitutes a well-characterized neurophysiological tool for comparing these modes of stimulation. Having evolved conceptually from a mere index of change detection to a marker of hierarchical predictive coding, the MMN provides a means to assess whether the brain treats two inputs as belonging to similar predictive models or as distinct categories. However, claims of equivalence require appropriate equivalence testing with a priori defined bounds and adequate statistical power, rather than a non-significant p-value alone. Moreover, measuring MMN in this context requires more than the classical oddball paradigm, which confounds prediction error with sensory adaptation. Among the available alternatives, the multi-feature paradigm is likely the most popular in the auditory field due to its efficiency, yet it remains susceptible to the same adaptation confound as the classical oddball. Therefore, studies employing roving, omission, and local–global paradigms are necessary to isolate genuine prediction error responses.

CI research has documented systematic, feature-specific deficits in electrically evoked predictive processing, but these findings cannot dissociate device-related limitations from hearing impairments. While the healthy somatosensory system can resolve this ambiguity, research in this field remains critically underdeveloped. Direct comparisons of natural versus electrical touch have only recently emerged due to a lack of widely available tactile stimulators compatible with neuroimaging methods. Limited evidence suggests electrotactile stimulation generates mismatch responses, but the cortical circuitry may differ from natural touch, which appears to engage more extensive bilateral integration, a conclusion drawn from a single MEG study and awaiting confirmation. These early findings underscore the need for further work, as such comparisons would position somatosensory research as a potential benchmark for understanding device-related limitations in the field of auditory neuromodulation and, potentially, other sensory domains.
